# Sleep apnea is associated with reduced in-hospital mortality in patients admitted for acute heart disease

**DOI:** 10.1371/journal.pone.0333797

**Published:** 2025-10-07

**Authors:** Maurice Moser, Florent Baty, Gian-Reto Kleger, Micha T. Maeder, Hans Rickli, Otto D. Schoch, Martin H. Brutsche

**Affiliations:** 1 Lung Center, Cantonal Hospital St. Gallen, St. Gallen, Switzerland; 2 Faculty of Medicine, University of Basel, Basel, Switzerland; 3 Department of Intensive Care, Cantonal Hospital St. Gallen, St. Gallen, Switzerland; 4 Department of Cardiology, Cantonal Hospital St. Gallen, St. Gallen, Switzerland; Cairo University Kasr Alainy Faculty of Medicine, EGYPT

## Abstract

**Background:** Sleep apnea (SA) is an independent risk factor for many cardiovascular diseases. The impact of SA on the outcome of patients hospitalized due to cardiovascular diseases is controversial. Recent studies indicated fewer fatal cardiac events associated with SA in a setting where co-factors may play an important role. The aim of the current study was to investigate in-hospital mortality of patients hospitalized with acute heart diseases with or without SA.

**Methods and findings:** In this retrospective, nested case-control study, data were extracted from a Swiss-wide hospitalization database. All patients with a primary diagnosis of acute heart disease were identified. Among those patients, all patients with a co-diagnosis of SA were extracted together with a control population matching the cases 1:1 for age, gender, month of hospitalization and Charlson’s comorbidity index. The impact of SA and other comorbidities was investigated using competing risks survival analysis. Between 2010 and 2020, 744,455 hospitalizations occurred with a primary diagnosis of acute heart disease in Switzerland among which 21,904 had a SA co-diagnosis. Patients with SA had a longer length of stay, were more often rehospitalized and had a higher number of comorbidities. On the other hand, they had a lower in-hospital mortality compared to controls (1.9 [1.8 to 2.1]% vs. 3.9 [3.7 to 4.2]%; subdistribution hazard ratio: 0.40 [95% confidence interval: 0.35 to 0.45], *p* < 0.001). Several comorbidities significantly interacted with SA. Obesity and hypertension were associated with a lower in-hospital mortality in both SA and non-SA. Shock was more frequent and significantly more harmful in non-SA patients.

**Conclusions:** A SA co-diagnosis in patients hospitalized due to acute heart disease was associated with a lower in-hospital mortality. Explanations for this survival paradox include the interplay of interacting comorbidities and hypoxic preconditioning contributing to the protective effect of SA.

## Introduction

Sleep apnea (SA) refers to brief, often periodic, interruptions in breathing accompanied with transient or sustained reductions in breathing amplitude resulting in oxygen desaturations, activation of the vegetative nervous system and fragmented sleep [1,2]. The reactive oxygen production due to intermittent hypoxia may trigger chronic inflammation often associated with other inflammatory disorders such as nasal chronic inflammation or metabolic syndrome [3,4]. Higher body mass index, diabetes, older age and male gender are independently associated with SA [5]. Sleep apnea is still an underdiagnosed disease. The prevalence in the general population is estimated to be higher than the diagnosed cases might indicate [6].

Many cardiovascular diseases, including coronary artery disease in general, myocardial infarction, arterial hypertension, cardiac arrhythmias or stroke are independently associated with SA [7–10]. The impact of SA on the outcome of patients hospitalized due to cardiac events is controversial, despite the fact that the association with cardiovascular diseases is widely established. Several studies have linked SA to a higher incidence of cardiovascular events and a lower long-term survival [11,12]. Nonetheless, more recent studies indicated a lower case-fatality rate in SA patients hospitalized with acute coronary syndromes [13,14]. In a recent study, a survival benefit was found in patients hospitalized with a known secondary diagnosis of SA [15]. The reasons for a protective effect of SA on cardiovascular events occurring during a hospital stay are not thoroughly understood and further research is needed. Possible explanations have been proposed in a number of human and animal studies, including the beneficial effects of intermittent hypoxia that occurs in patients with SA [16–19]. Other factors including the “obesity paradox” or an optimal therapy adjustment seem to correlate with an improved prognosis [20,21]. However, many of these factors underlying this paradox are still not fully understood. A comprehensive understanding of the interactions between SA, cardiovascular diseases and other comorbidities may help to clarify this paradox.

The aim of the current study was to further investigate the impact of SA on in-hospital mortality of patients hospitalized with acute heart diseases using a nation-wide hospitalization database, paying particular attention to the role played by interacting comorbidities.

## Materials and methods

### Swiss hospitalization database

Data were extracted from a nation-wide hospitalization database provided by the Swiss Federal Statistical Office. All hospitalizations in Switzerland were recorded in the database. Diagnoses were coded using the German modification of the International Classification of Diseases version 10 (ICD-10-GM). Patient information was fully anonymized and no written consent was required. No ethical approval was required for the current retrospective study. The database included 15,543,489 hospitalizations in the period between January 1^st^, 2010 and December 31^st^, 2020 (11 years). Each patient had a unique anonymous identifier. Information included year and month of hospitalization, age, sex, length of hospital stay (LOS), in-hospital mortality, primary diagnosis (reason for hospitalization) and up to 50 co-diagnoses.

### Nested case control design

The current study follows a nested case-control design. At first, all hospitalizations with a primary diagnosis of acute heart diseases were extracted from the database. The different acute heart diseases included the following subgroups: hypertension (I11.*, I132, I15.*, I16.*), myocardial infarction (I20.*, I21.*, I24.*, I2511, I2512, I2513, I255.*, I252.*, I253.*, I258.*), cardiomyopathy (I278.*, I279.*), heart failure (I501.*, I509.*), pulmonary heart disease (I278.*, I279.*), rhythm disorder (I44.*, I45.*, I46.*, I480.*, I4811, I4819, I49.*), valve disorder (I340.*, I348.*, I35.*, I36.*, I37.*), bypass/implants/pacemaker (Z950.*, Z951.*, Z952.*, Z953.*, Z954.*, Z955.*, Z9581, Z9582, Z959.*), and other heart diseases (I517.*, I52.*). The SA cases corresponded to patients hospitalized due to heart disease and a co-diagnosis of SA (ICD-10-GM codes G473.*). The control population consisted of patients hospitalized with a primary diagnosis of acute heart disease without SA co-diagnosis. In a 1:1 nested cases control design, the controls matched the cases for age, gender, month of hospitalization and Charlson’s comorbidity index. The nested-control population was obtained from the database using a random extraction procedure.

### Statistical considerations

Patient’s baseline characteristics were summarized using descriptive statistics. The effect of SA and other co-factors (including comorbidities) on patient’s time to in-hospital mortality was investigated using competing risks survival analysis [22,23]. In this study, recovery was considered as a competing risk for mortality which needs to be treated as an extra outcome instead of being censored like in standard survival analysis [24]. Plots of cumulative incidence functions were represented, and Gray’s test statistics were reported. Conditional logistic regression, was used to identify comorbidities significantly over- or under-represented in the SA cases. Results were reported as odds-ratio together with 95% confidence intervals and associated *p*–values. Comorbidities of interest were selected based on their significance levels (adjusted *p*–value < 0.05) and their prevalence.

The combined effect of SA with SA-associated comorbidities on in-hospital mortality was investigated using Fine-Gray risk regression models [25]. The proportional hazards assumption was checked by an evaluation of the Schoenfeld residuals. The interactive effect of the comorbidities was reported using subdistribution hazard ratios (HR_SD_) with 95% confidence intervals and associated *p*–values.

All analyses were done using the R statistical software (v. 4.3.1) [26] including the extension packages survival [27], cmprsk [28] and survminer [29].

## Results

### Hospitalizations for acute heart disease

**Fig 1:**
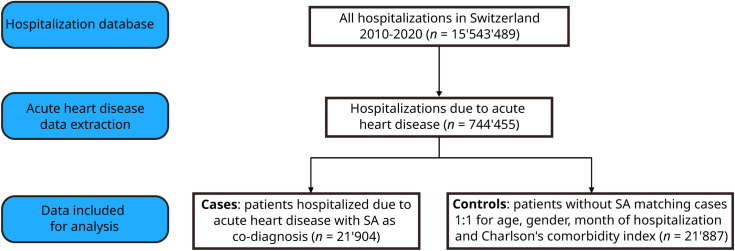
Flow diagram of the study protocol. The study has a nested case-control design.

The flow diagram of the current study protocol is shown in Fig 1. Between 2010 and 2020, the number of hospitalizations with a primary diagnosis of an acute heart disease was 744,455. Among these hospitalizations, 21,904 (2.9%) had a SA co-diagnosis (SA cases). The SA diagnoses included 6% of central SA (G4730), 68% of obstructive SA (G4731), 1% of sleep-related hypoventilation (G4732), 24% of other or unspecified SA (G4738 and G4739, respectively). Among the SA cases, 1.9% were on non-invasive ventilation. The baseline characteristics of the SA cases and matched controls without SA are summarized in Table 1. Compared to the controls, SA cases had a longer length of stay, a higher number of comorbidities, were more often re-hospitalized but had a lower rate of in-hospital mortality.

**Table 1:** Patients characteristics.

	Heart disease with SA (cases)	Heart disease without SA (controls)	*p*–val
Number of hospitalizations (*n*)	21,904	21,887	-
Number of unique patients	16,056	21,086	-
Age, mode of the distribution	70-74	70-74	-*
Sex ratio, males/females	81%	81%	-*
Median Charlson’s comorbidity index [IQR]	2 [1 to 3]	2 [1 to 3]	-*
Median length of stay in days [IQR]	7 [2 to 14]	5 [2 to 11]	<0.001
Median number of comorbidities [IQR]	10 [7 to 14]	7 [4 to 11]	<0.001
Rate of in-hospital mortality, % [95% CI]	1.9 [1.8 to 2.1]	3.9 [3.7 to 4.2]	<0.001
matched			

The prevalence of the heart diseases coded as primary reason for hospitalization is summarized in Table 2. The most frequent reasons for hospitalization were heart failure (20%), acute myocardial infarction (20%) and chronic ischemic heart disease (18%) in the SA cases and acute myocardial infarction (34%), chronic ischaemic heart disease (17%) and heart failure (14%) in non-SA patient.

**Table 2:** Main reasons for hospitalization. The prevalence of heart diseases are provided for both cases and controls as percentages.

Heart disease (primary ICD-10-GM code)	Prevalence cases, % [95% CI]	Prevalence controls, % [95% CI]	*p*–val
Hypertensive heart disease (I11)	4.4 [4.2 to 4.7]	2.2 [2.0 to 2.4]	<0.001
Hypertensive heart and chronic kidney disease (I13)	0.2 [0.2 to 0.3]	0.1 [0.1 to 0.2]	0.026
Secondary hypertension (I15)	0.1 [0.0 to 0.1]	0.1 [0.0 to 0.1]	0.712
Angina pectoris (I20)	7.7 [7.3 to 8.1]	8.1 [7.8 to 8.5]	0.082
Acute myocardial infarction (I21)	19.9 [19.3 to 20.4]	33.5 [32.9 to 34.1]	<0.001
Other acute ischemic heart diseases (I24)	0.3 [0.3 to 0.4]	0.5 [0.4 to 0.6]	0.018
Chronic ischemic heart disease (I25)	18.0 [17.5 to 18.5]	17.4 [16.9 to 17.9]	0.117
Other pulmonary heart diseases (I27)	0.5 [0.4 to 0.6]	0.2 [0.1 to 0.2]	<0.001
Nonrheumatic mitral valve disorders (I34)	2.6 [2.4 to 2.8]	2.2 [2.0 to 2.4]	0.003
Nonrheumatic aortic valve disorders (I35)	9.4 [9.0 to 9.8]	7.4 [7.1 to 7.8]	<0.001
Nonrheumatic tricuspid valve disorders (I36)	0.2 [0.1 to 0.3]	0.1 [0.1 to 0.1]	0.006
Nonrheumatic pulmonary valve disorders (I37)	0.0 [0.0 to 0.1]	0.0 [0.0 to 0.1]	1
Cardiomyopathy (I42)	2.4 [2.2 to 2.6]	1.6 [1.4 to 1.8]	<0.001
Atrioventricular and left bundle-branch block (I44)	2.4 [2.2 to 2.6]	2.1 [1.9 to 2.3]	0.048
Other conduction disorders (I45)	0.2 [0.1 to 0.3]	0.3 [0.2 to 0.4]	0.088
Cardiac arrest (I46)	0.4 [0.3 to 0.5]	0.5 [0.5 to 0.7]	0.011
Paroxysmal tachycardia (I47)	3.1 [2.9 to 3.3]	2.9 [2.7 to 3.1]	0.306
Atrial fibrillation and flutter (I48)	5.3 [5.0 to 5.6]	4.4 [4.2 to 4.7]	<0.001
Other cardiac arrhythmias (I49)	2.9 [2.7 to 3.2]	2.3 [2.1 to 2.5]	<0.001
Heart failure (I50)	19.9 [19.4 to 20.5]	13.9 [13.4 to 14.3]	<0.001
Complications and ill-defined descriptions of heart disease (I51)	0.1 [0.1 to 0.2]	0.1 [0.1 to 0.2]	0.402
Presence of cardiac and vascular implants and grafts (Z95)	0.0 [0.0 to 0.1]	0.0 [0.0 to 0.1]	0.452

The most frequent primary diagnoses associated with in-hospital mortality were heart failure (36%), acute myocardial infarction (21%) and cardiac arrest (7%) in the SA cases and acute myocardial infarction (44%), heart failure (23%) and cardiac arrest (8%) in the non-SA.

### Impact of SA on in-hospital mortality

**Fig 2:**
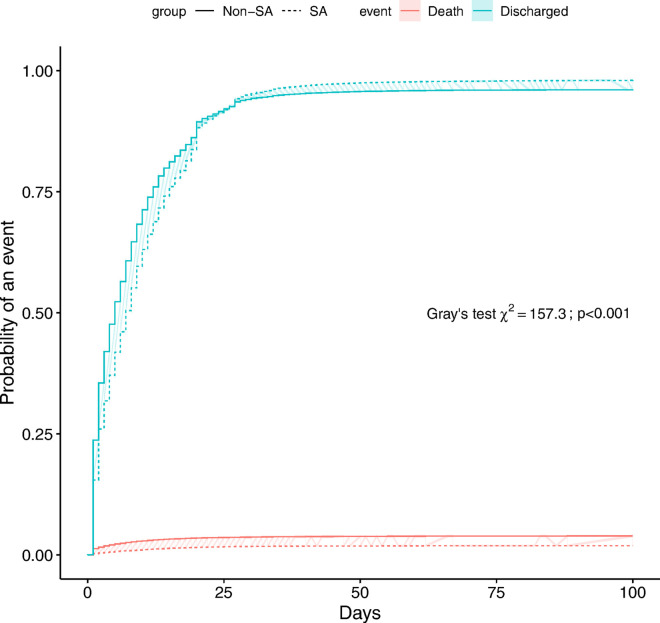
Cumulative incidence plot of in-hospital death and discharge in patients hospitalized for acute heart disease with or without SA. Gray’s test indicated a significant difference between the 2 groups for both death (*p* < 0.001) and recovery (*p* < 0.001).

Overall, the rate of in-hospital mortality was 1.9% (95% CI: 1.8 to 2.1) in SA cases and 3.9% (95% CI: 3.7 to 4.2) in controls. The in-hospital mortality did not significantly differ depending on the SA subtype (central vs. obstructive) ($\chi_{1}^{2}=2.29$; *p* = 0.131). SA apnea under non-invasive ventilation had a significantly higher in-hospital mortality ($\chi_{1}^{2}=9.08$; *p* = 0.003). In the competing risk analysis, SA was associated with a significant lower in-hospital mortality (HR_SD_: 0.4, 95% CI: 0.35 to 0.45; *p* < 0.001). The cumulative incidence function for in-hospital mortality and recovery (death/discharge) is shown in Fig 2. In controls, the estimated probability for in-hospital death was 2.9%, 3.8% and 3.9% during the first 10, 40 and 100 days of hospitalization, respectively. In SA cases, the estimated probability for in-hospital death was 1.1%, 1.8% and 1.9% during the first 10, 40 and 100 days of hospitalization, respectively.

### SA-associated comorbidities and in-hospital mortality

**Fig 3:**
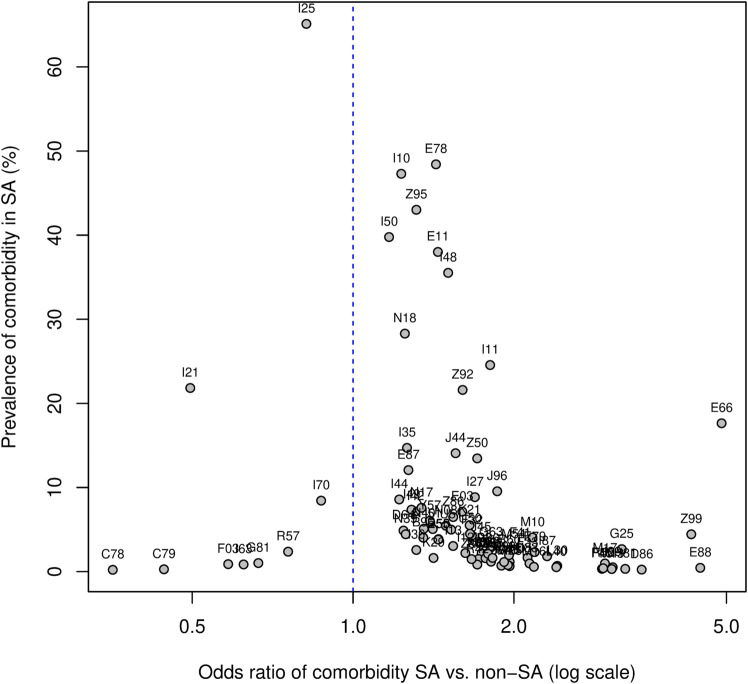
Comorbidity plot in SA patients hospitalized due to acute heart disease compared to matched controls. The prevalence of the comorbidities (y-axis) is reported as a function of the odds-ratio (x-axis). Comorbidities are displayed using 2-digit ICD-10-GM codes.

Overall, 85 comorbidities were significantly associated with SA, including 76 comorbidities over-represented and 9 under-represented in SA compared to controls (see S1 Table). Fig 3 depicts prevalence vs. odds ratio of comorbidities associated with SA. Comorbidities over-represented in SA cases included endocrine, nutritional and metabolic disorder such as obesity (E66), dyslipidemias (E78), type 2 diabetes mellitus (E11) and disorders of plasma-protein metabolism (E88). Heart conditions over-represented in SA-cases included hypertension (I10), hypertensive heart disease (I11), heart failure (I50) and atrial fibrillation and flutter (I48). On the other hand, acute myocardial infarction (I21), cerebral infarction (I63) and shock (R57) were under-represented in SA-cases compared to the controls.

**Fig 4:**
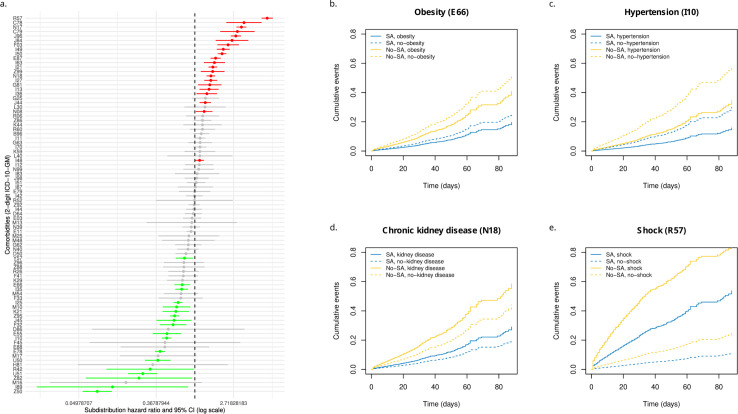
Subdistribution hazard ratios of SA-associated comorbidities and parametric estimates of cumulative incidence functions of four selected comorbidities. The subdistribution hazard ratios (HR_SD_) of each SA-associated comorbidities are represented together with the 95% confidence intervals in panel a. Comorbidities significantly associated with a higher in-hospital mortality (HR_SD_ >1) are shown in red whereas comorbidities significantly associated with lower in-hospital mortality (HR_SD_ <1) are represented in green. The cumulative incidence functions depicting the effects and interactions of four selected comorbidities are shown in panels b to e.

The impact of the 85 SA-associated comorbidities on in-hospital mortality is shown in Fig 4 (panel a). Comorbidities associated with a higher in-hospital mortality included shock (R57), secondary malignant neoplasm of respiratory, digestive organs and other unspecified sites (C78, C79), acute renal failure (N17), chronic kidney disease (N18), respiratory failure (J96), interstitial pulmonary diseases (J84), acute myocardial infarction (I21), cardiac arrhythmias (I49), heart failure (I50), cerebral infarction (I63), dementia (F03), extrapyramidal and movement disorders (G25) or disorders of fluid, electrolyte and acid-base balance (E87). Notable comorbidities associated with lower in-hospital mortality were essential (primary) hypertension (I10), chronic ischemic heart disease (I25), aortic valve disorders (I35), presence of electronic cardiac devices (Z95), gastro-esophageal reflux disease (K21), depressive episodes (F32), gout (M10), obesity (E66), disorders of lipoprotein metabolism (E78), asthma (J45) or care involving use of rehabilitation procedures (Z50). The effect and interaction of four illustrative comorbidities together with SA is presented in Fig 4 (panels b-e). Obesity and hypertension were independently associated with a significantly reduced in-hospital mortality in both SA and non-SA (HR_SD_: 0.72, 95% CI: 0.59 to 0.88; *p* < 0.001 and 0.48, 95% CI: 0.43 to 0.55; *p* < 0.001, respectively) (Fig 4, panels b and c). In contrast, chronic kidney diseases were associated with a poorer survival in both SA cases and non-SA (HR_SD_: 1.51, 95% CI: 1.36 to 1.69; *p* < 0.001) (Fig 4, panel d). Shock was also significantly associated with a poorer survival in both SA and non-SA (HR_SD_: 6.48, 95% CI: 5.62 to 7.49; *p* < 0.001). However, shock was less frequent (OR: 0.75, 95% CI: 0.67 to 0.85; *p* < 0.001) and tended to have a less unfavorable impact on the in-hospital mortality of SA compared to non-SA (interaction HR_SD_: 0.78, 95% CI: 0.59 to 1.04; *p* = 0.092) (Fig 4, panel e).

## Discussion

In cases with comorbid SA hospitalized for acute heart disease, we observed a significantly reduced in-hospital mortality when compared to controls without SA. This finding of a “sleep apnea survival paradox” is consistent with some previous studies which documented the connection between SA and an improved in-hospital survival in patients hospitalized due to heart diseases including acute myocardial infarction [12–14]. In addition, our study showed that several comorbidities were independently associated with a survival benefit. However, our results also suggested that the effect of SA on the in-hospital mortality was independent of key comorbidities such as obesity or hypertension. Finally, our analysis also highlighted the interacting effect of SA when combined with specific conditions including shock, cerebral and myocardial infarction.

A cohort study of Mokhlesi et al. also came to the conclusion that SA-patients undergoing elective cardiovascular, abdominal and orthopedic surgery had a significant lower rate of postoperative in-hospital mortality [30]. Another large retrospective cohort study with hospitalized pneumonia patients with SA showed lower in-hospital mortality, however they had an increased probability being transferred to the intensive care unit, longer length of stay and higher treatment costs [31]. Thus, the survival paradox seems to hold in different life-threatening scenarios.

To explain the paradox some studies postulate that SA patients are more likely to have active treatments like thrombolysis, percutaneous coronary intervention, and coronary- bypass operation due to the higher comorbidity profile and relatively younger age [13,32]. Additionally, there is a greater likelihood that SA-patients will be transferred to referral centers, which may be better equipped/staffed to handle more complex patients. Another possible explanation is that SA is frequently associated with obesity. Obese patients tend to have better in-hospital survival compared to non-obese through the so-called “obesity paradox” [33]. It is assumed that overweight patients show earlier cardiovascular symptoms and benefit from an optimized medical treatment [34,35]. Other effects such as tumor necrosis factor alpha (TNF*α*), which is increased in obese patients, also seems to play a role in the pathophysiology [36]. However our data show that obesity had an independent protective effect in patients with acute heart diseases with or without SA. It was already showed that obesity had a protective effect which is stronger in non-SA compared to SA [15]. Therefore, it can be assumed that obesity may not be the predominant explanation for the observed survival advantage in SA. Another assumption claims that SA-associated hypoxic preconditioning might be beneficial [13,19,37–39]. Lavie and colleagues hypothesized cerebro-protective mechanisms due to the oxidative stress associated with intermittent hypoxia [32]. Several studies postulate a protective effect of chronic intermittent hypoxia in the context of cardiac diseases [13,18,40,41]. Laboratory studies performed on rats previously exposed to intermittent hypoxia resulted in a decreased infarct size after occlusion [41]. Intermittent hypoxia induces neovascularization due to higher secretion of endothelial growth factor resulting in increased capillary density [18]. Increased collateral vessels in SA patients were also discovered in human studies [42]. This might also explain why SA patients show decreased troponin T-levels when suffering from acute myocardial infarction [16]. Ozeke and colleagues even go a step further and postulate that the survival benefit in obese individuals, i.e. “obesity paradox”, may actually be driven by hypoxic preconditioning induced by SA-associated chronic intermittent hypoxia, thus, the “SA survival paradox” [43].

Our study has several limitations. As the current study was performed retrospectively, no causality can be inferred. Only coded data on hospitalizations was available. The data is subject to coding quality in general and for acute heart diseases and SA in particular. Potential selection biases might have occurred by coding only severe symptomatic SA cases. In addition, patient data (e.g. BMI), SA-related diagnostic (e.g. diagnostic modality, SA severity, baseline O_2_) and treatment information – such as the use of continuous positive airway pressure (CPAP) – were not available. Based on our own experience, we can assume that the majority of patients were offered a CPAP treatment at home. Severity of SA and/or treatment status before and during the hospitalization may have influenced the outcome. Further investigation needs to be done to confirm the current findings.

## Conclusion

Our study confirms that patients hospitalized for acute heart disease with a known concomitant SA have a significant in-hospital survival advantage. This survival paradox could be explained by protective mechanisms around obesity and hypoxic preconditioning. The interplay of SA-associated comorbidities, however, can only partially explain the observed effect.

## Supporting information

S1 Table List of comorbidities significantly under- and over-represented in SA cases compared to matched controls without SA. The ICD-10-GM codes are provided together with the prevalences, odds ratios and *p*–values.
(PDF)
