# Supplementary material for: Sleep apnea is associated with reduced in-hospital mortality in patients admitted for acute heart disease
Source: PLoS One. 2025 Oct 7;20(10):e0333797. doi: 10.1371/journal.pone.0333797 (PMC12503258; doi:10.1371/journal.pone.0333797)
Supplement: S1 Table — The ICD-10-GM codes are provided together with the prevalences, odds ratios and p–values. (PDF) [file pone.0333797.s001.pdf]

Sleep apnea diagnosis and in-hospital mortality of patients with acute heart disease

Maurice Moser, Florent Baty, Gian-Reto Kleger, Micha T. Maeder, Hans Rickli, Otto D. Schoch, Martin H. Brutsche

Supplementary information

S1 Table

List of comorbidities significantly under- and over-represented in SA cases compared to matched controls without SA. The ICD10-GM codes are provided together with the prevalences (%), odds ratios and $p-$values.

ICD10	Details	Prevalence (%)	Odds-ratio	$p-$val
B96	Other specified bacterial agents as the cause of diseases	4.03	1.35	< 0.001
C78	Secondary malignant neoplasm of respiratory and digestive organs	0.21	0.35	< 0.001
C79	Secondary malignant neoplasm of other and unspecified sites	0.27	0.44	< 0.001
D50	Iron deficiency anaemia	3.86	1.45	< 0.001
D64	Other anaemias	4.88	1.24	< 0.001
D86	Sarcoidosis	0.24	3.47	< 0.001
E03	Other hypothyroidism	7.13	1.60	< 0.001
E11	Diabetes mellitus, Type 2	38.00	1.44	< 0.001
E55	Vitamin D deficiency	3.81	1.44	< 0.001
E66	Obesity	17.64	4.90	< 0.001
E78	Disorders of lipoprotein metabolism and other lipidaemias	48.42	1.43	< 0.001
E79	Disorders of purine and pyrimidine metabolism	2.32	2.19	< 0.001
E87	Other disorders of fluid, electrolyte and acid-base balance	12.07	1.27	< 0.001
E88	Other metabolic disorders	0.45	4.47	< 0.001
F03	Unspecified dementia	0.89	0.58	< 0.001
F32	Depressive episode	4.29	1.67	< 0.001
F33	Recurrent depressive disorder	1.65	2.12	< 0.001
F41	Other anxiety disorders	2.79	2.05	< 0.001
F45	Somatoform disorders	0.33	2.92	< 0.001
G25	Other extrapyramidal and movement disorders	2.67	3.18	< 0.001
G62	Other polyneuropathies	1.50	1.81	< 0.001
G63	Polyneuropathy in diseases classified elsewhere	2.88	1.81	< 0.001
G81	Hemiplegia	1.01	0.66	< 0.001
H81	Disorders of vestibular function	0.32	3.23	< 0.001
I10	Essential primary hypertension	47.29	1.23	< 0.001
I11	Hypertensive heart disease	24.56	1.81	< 0.001
I12	Hypertensive renal disease	2.21	1.62	< 0.001
I13	Hypertensive heart and renal disease	3.04	1.54	< 0.001
I21	Acute myocardial infarction	21.83	0.50	< 0.001
I25	Chronic ischaemic heart disease	65.12	0.82	< 0.001
I27	Other pulmonary heart diseases	8.85	1.69	< 0.001
I35	Nonrheumatic aortic valve disorders	14.71	1.26	< 0.001
I36	Nonrheumatic tricuspid valve disorders	2.57	1.31	< 0.001
I42	Cardiomyopathy	7.15	1.31	< 0.001
I44	Atrioventricular and left bundle-branch block	8.58	1.22	< 0.001
I48	Atrial fibrillation and flutter	35.52	1.51	< 0.001
I49	Other cardiac arrhythmias	7.36	1.29	< 0.001
I50	Heart failure	39.77	1.17	< 0.001
I51	Complications and ill-defined descriptions of heart disease	5.06	1.41	< 0.001
I63	Cerebral infarction	0.85	0.62	< 0.001
I70	Atherosclerosis	8.44	0.87	< 0.001
I83	Varicose veins of lower extremities	0.97	2.14	< 0.001
I87	Other disorders of veins	1.83	2.31	< 0.001
I89	Other noninfective disorders of lymphatic vessels and lymph nodes	0.52	3.06	< 0.001

J44	Other chronic obstructive pulmonary disease	14.08	1.56	< 0.001
J45	Asthma	3.47	1.74	< 0.001
J84	Other interstitial pulmonary diseases	0.71	1.90	< 0.001
J96	Respiratory failure, not elsewhere classified	9.55	1.86	< 0.001
J98	Other respiratory disorders	2.18	1.74	< 0.001
K21	Gastro-oesophageal reflux disease	5.51	1.65	< 0.001
K29	Gastritis and duodenitis	1.62	1.41	< 0.001
K44	Diaphragmatic hernia	1.17	1.96	< 0.001
K59	Other functional intestinal disorders	1.50	1.72	< 0.001
L30	Other dermatitis	0.68	2.41	< 0.001
L40	Psoriasis	0.53	2.40	< 0.001
M10	Gout	4.05	2.17	< 0.001
M13	Other arthritis	0.31	3.05	< 0.001
M16	Coxarthrosis [arthrosis of hip]	0.57	2.18	< 0.001
M17	Gonarthrosis [arthrosis of knee]	0.97	2.96	< 0.001
M25	Other joint disorders, not elsewhere classified	0.65	1.96	< 0.001
M48	Other spondylopathies	0.78	1.95	< 0.001
M54	Dorsalgia	2.63	2.00	< 0.001
N08	Glomerular disorders in diseases classified elsewhere	5.46	1.49	< 0.001
N17	Acute renal failure	7.59	1.34	< 0.001
N18	Chronic kidney disease	28.29	1.25	< 0.001
N39	Other disorders of urinary system	4.44	1.25	< 0.001
N40	Hyperplasia of prostate	5.10	1.36	< 0.001
N99	Postprocedural disorders of genitourinary system, not elsewhere classified	1.14	1.92	< 0.001
R06	Abnormalities of breathing	1.60	1.77	< 0.001
R26	Abnormalities of gait and mobility	2.11	1.79	< 0.001
R42	Dizziness and giddiness	0.84	1.71	< 0.001
R52	Pain, not elsewhere classified	0.40	2.94	< 0.001
R57	Shock, not elsewhere classified	2.37	0.76	< 0.001
R60	Oedema, not elsewhere classified	1.96	1.96	< 0.001
Y57	Other and unspecified drugs and medicaments	6.04	1.39	< 0.001
Z50	Rehabilitation	13.46	1.71	< 0.001
Z82	Disabilities, chronic illneses	1.18	1.81	< 0.001
Z86	Personal history of certain other illnesses	6.48	1.54	< 0.001
Z88	Allergy to medicines, drugs or biological	1.48	1.67	< 0.001
Z92	Medical treatment in personal history	21.60	1.60	< 0.001
Z95	Presence of cardiac or vascular implant	43.01	1.31	< 0.001
Z96	Presence of other functional implants	1.63	1.82	< 0.001
Z99	Dependence on supportive devices	4.44	4.30	< 0.001
U50	Motor function limitation	4.50	1.66	< 0.001
U51	Cognitive functional impairment	4.99	1.53	< 0.001
